# Cultural Roles of Native Patient Navigators for American Indian Cancer Patients

**DOI:** 10.3389/fonc.2015.00079

**Published:** 2015-04-30

**Authors:** Linda Burhansstipanov, Lisa Harjo, Linda U. Krebs, Audrey Marshall, Denise Lindstrom

**Affiliations:** ^1^Native American Cancer Research Corporation, Pine, CO, USA; ^2^Anschutz Medical Campus, College of Nursing, University of Colorado at Denver, Denver, CO, USA

**Keywords:** American Indian, native, patient navigators, cultural roles, community-based navigation services, cancer

The purpose of this opinion article is to clarify cultural roles Native Patient Navigators (NPNs) perform in providing cancer support. NPNs, who are American Indian (AI), provide many unique services to indigenous patients who are undergoing treatment (radiation, chemotherapy, surgery, adjuvant therapy) for cancer. AIs experiences of cancer often are complex, requiring a team that not only provides comprehensive, quality cancer care, but also provides care that incorporates cultural norms and beliefs. NPNs are an essential component of AI cancer patients’ recovery and healing.

American Indians in the USA have distinct and significant geographic rates of cancer incidence and mortality, whereas White rates remain homogeneous (1–5). Indigenous people living in Alaska and the Northern (e.g., ND, SD, NE, WI, MT, MI) and Southern Plains (e.g., OK, TX, KS) typically have elevated age-adjusted cancer incidence and mortality rates. The substantial progress in reducing cancer death rates experienced by Whites over the past two decades has not been experienced by AIs (6); cancer mortality rates remain the same or more commonly are increased from previous data (1–5). AIs continue to have the poorest 5-year relative survival from cancer in comparison to all other ethnic and minority groups in the US (66.7% for non-Hispanic Whites vs. 59.0% for AIs) (7, 8). Anecdotal data from Canadian First Nations or Aboriginals, New Zealand Maoris, and Australian Aboriginals report similar geographic variability in their respective countries.

According to Harold Freeman, MD, the “father” of patient navigation, navigators guide patients through and around barriers in the complex health care system, to help ensure timely diagnosis and treatment (9) of cancer and other illnesses. However, the term “navigator” has varied meanings within healthcare systems, resulting in some confusion. As an example, the federal Affordable Care Act (H.R. 3590) (10) refers to navigators as trained individuals who “establish relationships with employers and employees, consumers (including uninsured and underinsured consumers), or self-employed individuals likely to be qualified to enroll in a qualified health plan” (11). Thus, under the Act, a navigator functions mainly as an insurance broker rather than one who helps patients overcome barriers to accessing and using a specific healthcare system or treatment plan/program. Within many settings, Community Health Workers, who are culturally, well-respected members of underserved populations, help bring these community members to the doors of the clinic or healthcare facility. NPNs function similarly to Community Health Workers; however, they cross the threshold of the clinic and continue providing cultural support within clinical departments (i.e., they cross boundaries). NPNs are familiar with varying tribal beliefs about health and illness and can establish a rapport and trust with patients that allow them to share their fears and spiritual practices necessary to achieve health and healing. NPNs provide services and support that are unlikely to be addressed by other hospital staff and they need to be paid lay professional positions. Ideally, the NPN and the hospital collaborate to provide the optimal healing environment for the AI patients.

## Key Points

- NPNs should come from or be familiar with and trusted by the local community (reservations, rural or urban settings).
- NPNs in urban settings should be able to work with AIs who come from many different tribes.
- NPNs need to be respectful of the local cultures and have cross-cultural skills. All NPNs will encounter AIs from different tribes; because of trust relationships with local communities, inter-tribal cultural differences have not been an issue.
- NPNs use respect and communication skills to allow AI patients to share personal, cultural, and religious needs related to their health and possible treatment.
- NPNs are an extension of the medical community and provide services that are not duplicated within most settings. Therefore, they should be paid.

Native American Cancer Research Corporation (NACR) has paid NPNs since 1995. NACR NPNs have navigated more than 1,000 AIs. A few examples from NACR’s experienced demonstrate cultural roles as well as the collaboration with clinical staff. These roles may be comparable for indigenous people living in other parts of the US as well as countries outside the US.

1. A few patients asked the NPN to remove chairs from their hospital rooms. One patient told the NPN that in his weakened state of mind, spirits may come into the room and convince him to go with them. If the chairs were in the hallway, the spirits had no place to sit in the room. The NPN removed the chairs and placed them in the hallway; hospital staff kept moving them back. The NPN explained the patient’s tribal beliefs and requested the staff to leave the chairs in the hallway. The hospital staff was appreciative that the NPN shared this information. When the NPN returned the next morning, all of the chairs were in the hallway. Everyone wanted these patients to heal in a respectful manner and the patients appreciated the attention to their cultural beliefs and healing.
2. Most AIs believe that certain traditional items such as feathers, medicine bags, or stones have healing powers. NPN explained to clinical staff the importance of such items and asked the staff to assist the patient in keeping these items in close proximity. To support this patient’s belief, an oncology nurse suggested using Betadine to sterilize the outer upper thigh, placing the medicine bag inside a sterile bag, taping the bag to the sterilized area on the thigh, then applying Betadine over the entire area. Having the bag with the patient provided additional spiritual strength for healing, and the bag’s placement as suggested by the nurse was in an area that would not interfere with any procedures.
3. One of the local hospitals used an owl (showing a knowledgeable bird) on hallway walls to guide patients to specific treatment rooms (radiation, chemotherapy). However, to many AI Tribal Nations, the owl signifies that death is near. The NPNs explained this belief to hospital authorities who responded that there were too few AI patients for them to change the hallway symbols. To avoid the patient being exposed to the symbol, the NPNs escorted the patients to alternative routes.
4. Many AI Tribal Nations burn natural plants (e.g., sage, cedar, sweet grass) to clean the local environment or to remove spirits that may interfere with good feelings, attitudes, and medicines. This is called “smudging” and the plants are usually burned using a shell or rock. Several AI patients have requested to have the hospital room smudged to contribute to their healing and recovery. However, in the USA, most hospitals have smoke-free policies. NPNs worked with clinical staff and traditional healers to find compromises. The hospital staff and NPNs transported the patient to an outside area that was delineated for smokers. The patient and bed were smudged outdoors and the remaining leaves in the shell were returned to the hospital room and used as potpourri. In another example, the patient was too sick to take outside but the Chapel in the hospital allowed candles and incense. The NPN brought the patient to the Chapel to burn the sage.
5. NPNs understand that many Tribal Nations believe that hair should be saved throughout life. It may be used in a variety of ways including being placed in the pillow placed underneath the head when buried. Patients receiving chemotherapy frequently lose their hair. Thus, when cleaning a brush or showering, the hair should be retained and not thrown away. The NPNs explain the patients’ beliefs about not disposing hair to hospital staff and caregivers to provide support to the patient.

When considered alone, each of these examples are mere illustrations of cultural issues that NPNs address on behalf of their patients. Native navigation is not limited to these examples, but is a holistic approach to meeting the patients’ cultural needs.

## Conclusion

Native Patient Navigators play a key role in providing a supportive healing atmosphere for AI patients because they understand the culture and beliefs of the patients they serve. Healthcare staffs are dedicated to the healing and recovery of their patients and, to date, have almost always welcomed the cultural guidance provided by the NPNs. These NPNs need to receive salaries to support their invaluable expertise and skills in helping AI patients through the cancer experience. Healthcare providers can greatly assist cultural navigators to become eligible to receive payment for their services. One possible method to provide payment might be for the state departments of public health or insurance companies to pay organizations to hire a “Native Patient Navigator.” These NPNs can be on call by any and all of the identified cancer centers, dependent on the source of the funds, to assist AI patients with cancer move through the system in a respectful manner. This way, a culturally appropriate native person is available to meet the needs of AIs in varying institutions, and no one place has to foot the entire bill since there are not enough AI patients in any one cancer center at one time. Once navigators are approved through insurance as a reimbursable cost, the cost can be spread across many institutions. It could be used as a strategy for other cultural and ethnic groups as well, dependent on the location and the need. But to make this strategy work, the NPN needs to be a regular employee on a continuous basis and not month to month, or year to year.

What we have learned from AI NPNs is relevant to other populations that live with health inequities in order to improve patient care and outcomes. Other populations need to adapt their cultural components within their respective navigation programs and use cultural navigators, such as NPNs, to support health, healing, and recovery.

## Conflict of Interest Statement

The authors declare that the research was conducted in the absence of any commercial or financial relationships that could be construed as a potential conflict of interest. The Associate Editor Daniel Grant Petereit declares that, despite having collaborated with authors Linda Burhansstipanov, Lisa Harjo, and Linda U. Krebs, the review process was handled objectively and no conflict of interest exists.
